# On Diseases Peculiar to Women

**Published:** 1861-01

**Authors:** 


### BOOK AND PAMPHLET NOTICES.

On Diseases Peculiar to Women, including Displacements of the Uterus. By Hugh L. Hodge, M. D., Prof. of Obstetrics and Diseases of Women and Children, in the University of Pennsylvania, with Original Illustrations. Philadelphia, Blanchard & Lea, 1860.

This is a good sized octavo volume of 469 pages, done up in the best style of the well known publishers, Blanchard and Lea. It is divided into *three* parts. The first contains twelve chapters, devoted to a consideration of *Irritation* of the *Uterus*, its causes, symptoms, complications, consequences, and treatment. The second, embraces nine chapters on the various displacements of the uterus, and their treatment. The third, embraces three chapters on "Sedation of the Uterus." The author's style is clear, concise and pleasing; while the doctrines he inculcates are eminently practical. Every practitioner will find the work really a valuable addition to his library. It is for sale by Keen & Co., Booksellers, Chicago.

